# Semi-field studies to better understand the impact of eave tubes on mosquito mortality and behaviour

**DOI:** 10.1186/s12936-018-2457-4

**Published:** 2018-08-22

**Authors:** Antoine M. G. Barreaux, N’Guessan Brou, Alphonsine A. Koffi, Raphaël N’Guessan, Welbeck A. Oumbouke, Innocent Z. Tia, Matthew B. Thomas

**Affiliations:** 1 Center for Infectious Disease Dynamics & Department of Entomology, Pennsylvania State University, University Park, PA 16802 USA; 2 Institut Pierre Richet/Institut National de santé Publique (INSP), Bouaké, Côte d’Ivoire; 3 LondonSchool of Hygiene and Tropical Medicine, Keppel Street, London, UK; 4 Centre d’Entomologie Médicale et Vétérinaire, Université Alassane Ouattara, Bouaké, Côte d’Ivoire

**Keywords:** Vector control, Housing improvement, Mosquito entry, *Anopheles gambiae*, Blood-feeding inhibition, Deflection, Malaria

## Abstract

**Background:**

Eave tubes are a type of housing modification that provide a novel way of delivering insecticides to mosquitoes as they attempt to enter the house. The current study reports on a series of semi-field studies aimed at improving the understanding of how eave tubes might impact mosquito mortality and behaviour.

**Methods:**

Experiments were conducted using West African style experimental huts at a field site in M’be, Côte d’Ivoire. Huts were modified in various ways to determine: (i) whether mosquitoes in this field setting naturally recruit to eave tubes; (ii) whether eave tubes can reduce house entry even in the absence of screening; (iii) whether mosquitoes suffer mortality if they attempt to exit a house via treated eave tubes; and, (iv) whether screening and eave tubes might deflect mosquitoes into neighbouring houses without the intervention.

**Results:**

Ninety percent more mosquitoes (*Anopheles gambiae* sensu lato, and other species) entered huts through open eaves tubes compared to window slits. The addition of insecticide-treated eave tubes reduced mosquito entry by 60%, even when windows remained open. Those mosquitoes that managed to enter the huts exhibited a 64% reduction in blood feeding and a tendency for increased mortality, suggesting contact with insecticide-treated inserts prior to hut entry. When *An. gambiae* mosquitoes were deliberately introduced into huts with treated eave tubes, there was evidence of six times increase in overnight mortality, suggesting mosquitoes can contact treated eave tube inserts when trying to exit the hut. There was no evidence for deflection of mosquitoes from huts with screening, or screening plus eave tubes, to adjacent unmodified huts.

**Conclusions:**

Eave tubes are a potentially effective way to target *Anopheles* mosquitoes with insecticides. That treated eave tubes can reduce mosquito entry even when windows are open is a potentially important result as it suggests that eave tubes might not need to be combined with household screening to have an impact on malaria transmission. The absence of deflection is also a potentially important result as coverage of eave tubes and/or screening is unlikely to be 100% and it is important that households that do not have the technology are not disadvantaged by those that do.

**Electronic supplementary material:**

The online version of this article (10.1186/s12936-018-2457-4) contains supplementary material, which is available to authorized users.

## Background

It is generally accepted that new vector control tools are needed to assist in driving down malaria transmission and achieve the control targets set out in the World Health Organization (WHO) Global Technical Strategy [1–3]. Eave tubes have been proposed as a new tool for delivering insecticides to *Anopheles* mosquitoes as they search for hosts and attempt to enter houses to blood feed [4]. When combined with screening of doors and windows, preliminary evidence suggests that eave tubes reduce entry of mosquitoes and increase overnight mortality rate, leading to reduced transmission risk at both household and community levels [4–7].

The epidemiological impact of screening plus eave tubes is currently being evaluated in a large-scale cluster randomized trial (CRT) in 40 villages in central Côte d’Ivoire [8]. In parallel with this CRT, a number of small-scale studies are being conducted in Côte d’Ivoire to help better understand the functioning of screening and eave tubes and potentially assist in interpreting the ultimate impacts of the intervention on transmission. This paper reports on a series of experiments exploring the effects of screening and eave tubes on mosquito behaviour and mortality. The approach used West African style experimental huts to investigate: (i) whether mosquitoes in this field setting naturally recruit to eave tubes; (ii) whether eave tubes can reduce house entry even in the absence of screening; (iii) whether mosquitoes suffer mortality if they attempt to exit a house via treated eave tubes; and, (iv) whether screening and eave tubes might deflect mosquitoes into neighbouring houses without the intervention.

## Methods

### Mosquito populations

All studies were conducted in the experimental site of M’be (5.209963 W and 7. 970241 N), in central Côte d’Ivoire [9, 10]. The malaria vectors in this area are dominated by *Anopheles gambiae* sensu lato (*s.l*.) and are known to be highly resistant to pyrethroids [11–13]. Mosquitoes were reared before release and/or brought back for observation and analysis in laboratory at the Institut Pierre Richet (IPR) research centre in Bouaké, Côte d’Ivoire.

Mosquitoes were hand-captured one-by-one inside the experimental huts and enclosure using individual glass haemolysis tubes and a flash light. Tubes were plugged with a small piece of cotton and labelled, prior to transportation to the lab. Mosquitoes were then identified to species level using a binocular microscope (40×). The fact that they were alive or not, and blood fed or not was also assessed. Mosquitoes alive at capture (or recapture) were kept for observation for 24 h in the insectary on 10% honey solution, at 27 ± 2 °C, 60 ± 20% RH and ambient light. Their mortality was assessed after 24 h.

In experiments where mosquitoes were released (as opposed to recruiting naturally into experimental huts from the wild), experimental mosquitoes were derived from larval collections in the local area. These mosquitoes are known to be insecticide resistant [11–13]. The field-collected *An. gambiae* larvae were maintained at standard density (about 300 larvae) in metallic bowls with about 1 l of deionized water and fed daily with fish food (Tetramin™ baby) until pupation. Upon emergence, adult mosquitoes were housed in standard mosquito cages and maintained on 10% honey solution at 27 ± 2 °C, 60 ± 20% RH and ambient light.

### Experimental huts with eave tubes

**Fig. 1:**
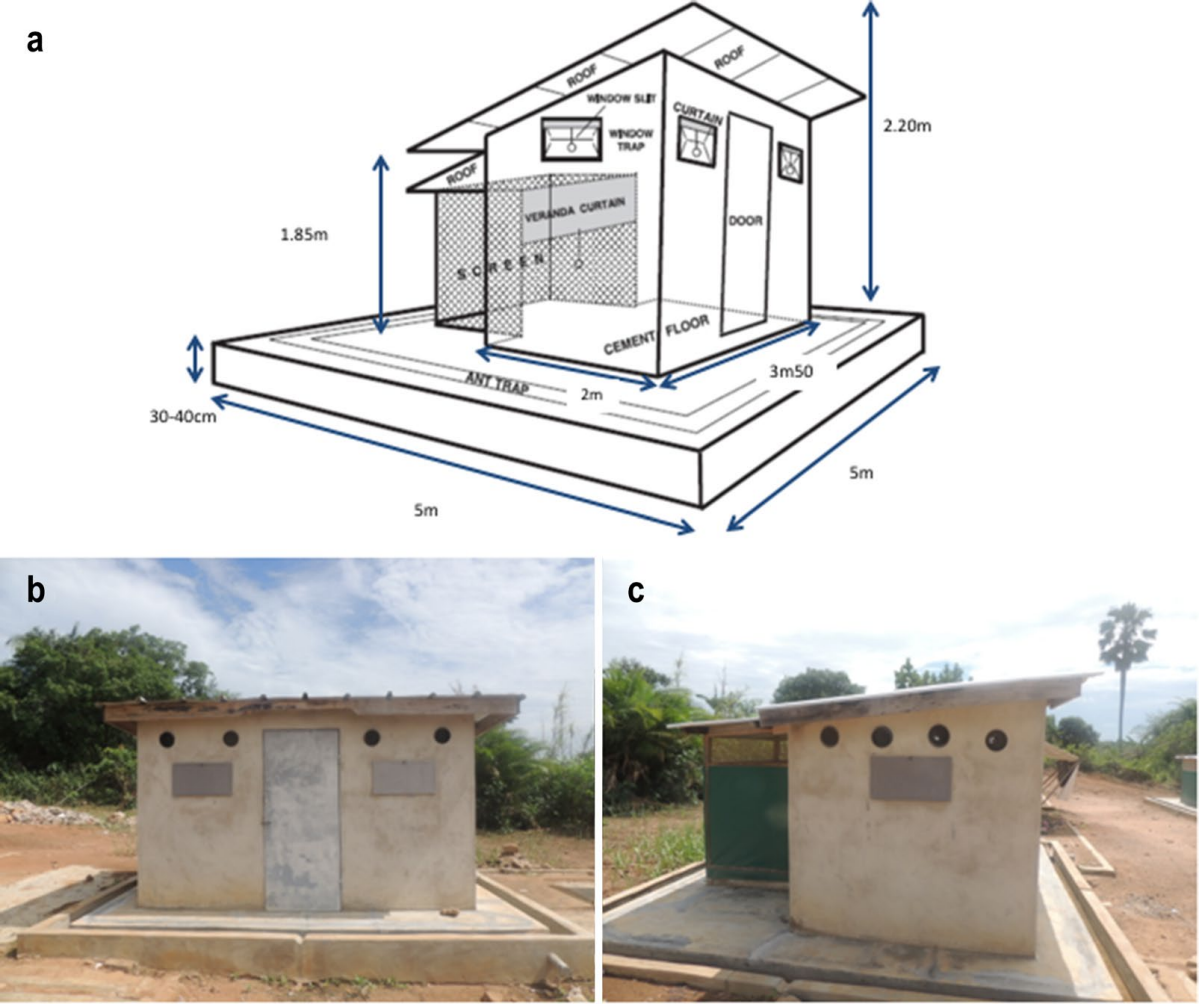
West African experimental hut in M’be, Côte d’Ivoire, and modifications with addition of eave tubes. **a** Is the schematic from the experimental hut (modified from Djènontin et al. [14]). **b** Represents the front of the hut, **c** the left side of the hut. The huts were modified to include multiple tubes (12) for use in other experiments but for the current study, half the tubes were blocked so that each experimental hut had 6 functioning eave tubes (2 on each side and 2 at the front)

**Fig. 2:**
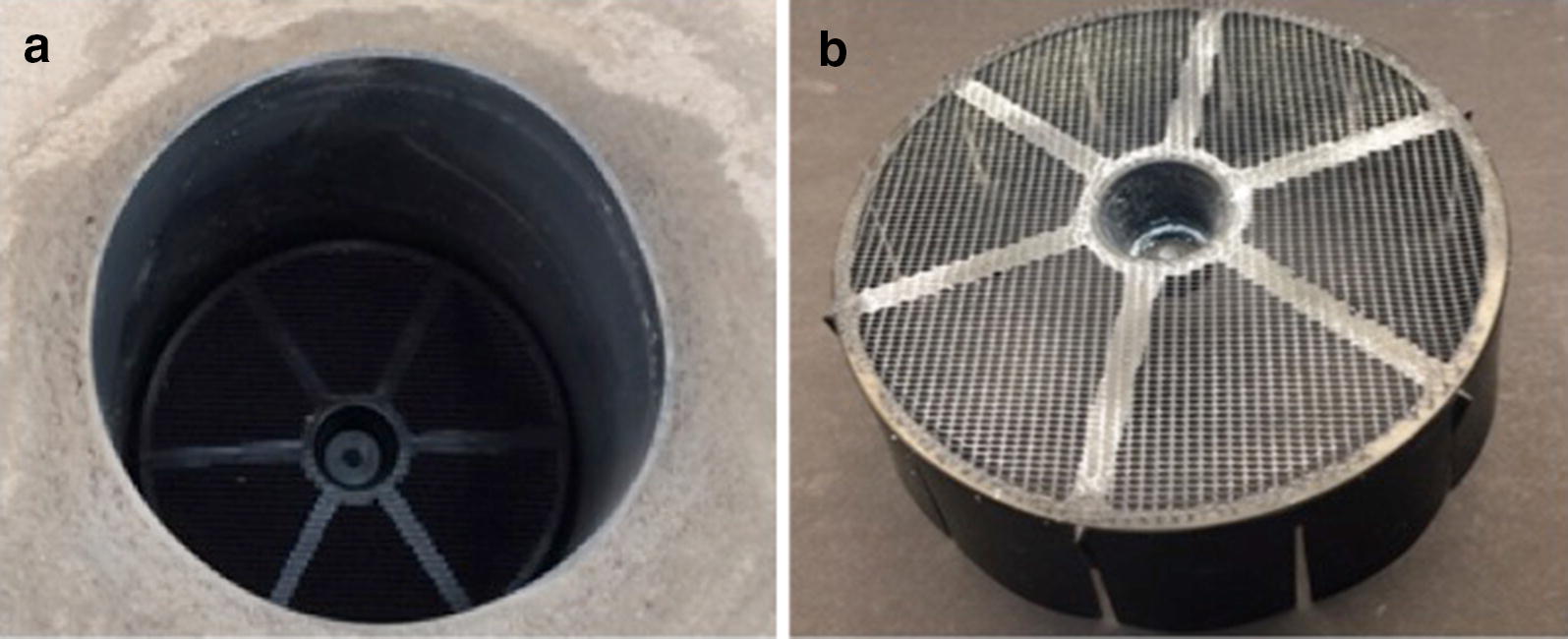
Eave tube and eave tube insert. **a** An insert inside an eave tube (view from outside); **b** a treated insert with visible insecticide powder

Eave tubes were installed in standard West African experimental huts [14, 15] by drilling 15-cm holes at eave level, at a 10° angle from the horizontal. Huts were modified to accommodate a total of 12 tubes per hut, but for the current study, half of the openings were blocked, and the remaining 6 tubes (two on each side and two at the front) were used as functional eave tubes (Fig. 1). A 20-cm long piece of polyvinyl chloride (PVC) pipe was fixed inside each hole to house the eave tube inserts (Fig. 2). As is typical for this type of experimental hut, each hut had four metallic windows with a horizontal window slit in each (two sheets of metal form a funnel inside the window frame with a narrow opening enabling mosquito entry but preventing mosquito exit) and a metallic shutter that can be closed.

### Eave tube inserts

The current approach for delivering insecticides in the eave tubes uses plastic inserts containing netting treated with an electrostatic coating. This coating provides a long-lasting static charge that enables insecticide powders to bind to the netting. The inserts block the entry of mosquitoes while contact with the netting leads to transfer of insecticidal particles onto the mosquito body [4, 5]. The dose transferred is sufficiently high that lethal doses of insecticide can be delivered during transient contact, even when the mosquitoes are classified as ‘resistant’ to the active ingredient [4, 5].

Untreated eave tube plastic inserts containing electrostatic netting were produced by In2Care^®^ in The Netherlands and then machine-treated with insecticide powder in Bouake, Côte d’Ivoire [16]. The inserts were treated with a wettable powder formulation of 10% beta-cyfluthrin (Tempo 10©, Bayer), which is the same product currently being used in the CRT in Cote d’Ivoire. The application procedure applies in the range of 300–500 mg of insecticide powder per insert (the surface of the insert is about 150 cm^2^).

### Sample size calculations

The number of replicates in the various experiments described below was determined in the first instance based on availability of mosquitoes, personnel and time. However, the replication was checked retrospectively based on the empirical data using the “pwr package” in R. For the primary read-outs of the experiments the number of sample nights was above the number required to demonstrate 5% significance with 70–80% power. For the deflection study, the number of nights was sufficient to detect a potential deflection effect of 50% and above.

### Experimental Designs

#### (i) Mosquito recruitment to eave tubes

An experiment was conducted to determine whether mosquitoes in the field naturally recruit to eave tubes. The approach used 2 experimental huts that were assigned one of two treatments: (i) open eave tubes and closed windows, or (ii) open windows and closed eave tubes. Each hut had a sleeper protected under a long-lasting insecticide-treated net (LLIN; Permanet 2.0©) to act as a host cue. The eaves tubes or the windows were left open to enable mosquitoes to recruit naturally through the eaves or the window slits. The sleepers entered the huts at 20.00 and the windows or the eaves were opened by the supervisor. At 05.00 the following morning, the windows or the eaves were closed by the supervisor (the experimental period from 20.00 to 05.00 is representative of the period when household members are likely to be indoors and is typical for experimental hut studies [9, 10]). The sleepers then collected all mosquitoes that had entered the huts overnight. Treatments were rotated between huts over a total of 20 nights so that each treatment was replicated at least ten times.

#### (ii) Mosquito entry through windows in the presence of eave tubes

The aim of this experiment was to determine whether eave tubes alone could impact mosquito entry, blood feeding rate and overnight mortality, even in the absence of ‘window screening’. The approach used 2 experimental huts that had either insecticide-treated or untreated inserts placed within the eave tubes and open windows at night. Each hut had a sleeper protected under an LLIN (Permanet 2.0©) to act as a host cue. The windows were left open to enable mosquitoes to recruit naturally through the window slits. The sleepers entered the huts at 20.00 and the supervisor opened the windows. The supervisor closed the windows of the huts at 05.00 the following morning and the sleepers collected all mosquitoes that had entered the huts overnight. Sleepers and treatments (i.e. treated or untreated inserts) were rotated between huts over a total of 24 nights, giving 6 replicates of each combination of hut, treatment and sleeper.

#### (iii) Exit mortality of mosquitoes

To evaluate whether insecticide-treated eave tubes might cause increased mortality of mosquitoes attempting to exit a house after a blood meal attempt, 4-5 days old non-blood-fed female *An. gambiae* mosquitoes were released inside two experimental huts, each with a sleeper protected under an LLIN. One hut was fitted with treated inserts and the other with untreated inserts (control). The windows and the curtain to the veranda were closed in the huts to prevent exit from the sleeping area. Sleepers entered the huts at 20.00 and a technician released the mosquitoes at 20.15. Mosquitoes were then collected back from the hut at 05.00. Fifty to 100 mosquitoes were released per night and the treatment was rotated between huts and sleepers for a total of 8 replicate nights.

The number of dead mosquitoes at recapture inside these huts with blocked exits was measured. Live mosquitoes were brought back to the laboratory and their mortality assessed 24 h post recapture.

The only difference between the hut fitted with treated eave tubes and the hut fitted with untreated eave tubes was the presence of insecticide on the eave tube inserts. Accordingly, any additional mortality of mosquitoes was attributed to mosquitoes contacting the treated inserts, presumably as they attempt to exit the huts, which is defined here as “exit mortality”.

#### (iv) Deflection of mosquitoes

The goal of this experiment was to determine if screening houses and adding eave tubes causes deflection of mosquitoes, potentially increasing the number of mosquitoes that enter neighbouring houses with no intervention.

**Fig. 3:**
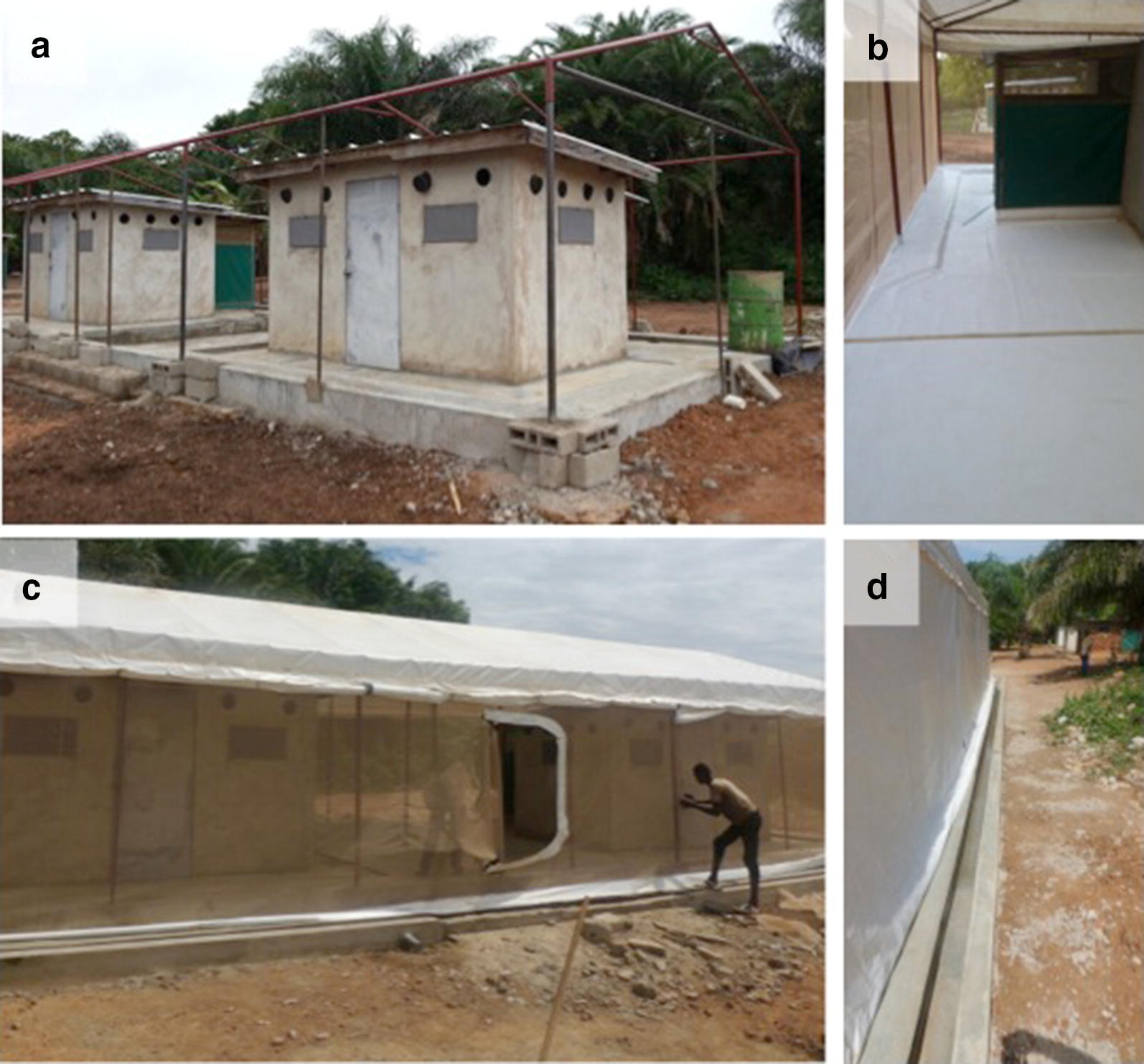
Semi-field enclosure for release-recapture studies. **a** Metallic framework of the enclosure built around 2 experimental huts; **b** white tarpaulin floor to facilitate collection of dead mosquitoes; **c** netting walls and door, and tarpaulin roof; **d** water gutter to reduce entry of ants

To explore risk of deflection it was necessary to erect a large screen house (5 m wide, 13 m long and about 4 m high) to enclose 2 experimental huts (Fig. 3). Huts were assigned 1 of 3 treatments: (i) control, in which windows and eave tubes were open; (ii) screened, in which windows were closed and eave tubes were closed with untreated inserts; and, (iii) treated eave tubes, in which windows were closed and the eave tubes contained insecticide-treated inserts. In all cases, the doors of the huts were closed, and a sleeper was present in each hut, protected by an untreated bed net to avoid any potential repellence effects.

The treatments were paired in the following way: control + control, control + screened, and control + treated eave tubes. The treatments and sleepers were rotated over the 2 huts with a total of 24 releases and 8 replicates of each combination of hut treatments. For each release night, 90–100 female *An. gambiae* were released in the central area of the enclosure at 20.15 (Fig. 3). The mosquitoes were 4–5 days old non-blood fed females that were starved for 6 h before release. Mosquitoes were then collected back in at 05.00 the next morning, with their position recorded (i.e., whether they were inside one or other hut, or outside the huts in the enclosure).

### Analysis

#### Mosquito entry through open eaves or windows

The number of *An. gambiae* mosquitoes captured was analysed using a linear mixed model that included the hut treatment (open windows or eaves) as independent variable. The night of capture and the hut were considered as random effects.

The same analysis was conducted for the total number of mosquitoes captured

The data were log transformed to fit a normal distribution for both analyses.

#### Mosquito entry comparing treated and untreated inserts

The number of mosquitoes captured was analysed using a linear mixed model that included insert treatment as independent variable. The night of capture, the hut and the sleeper were considered as random effects. The same analysis was done for the blood feeding rate, mortality at recapture, and mortality 24 h post recapture.

The blood-feeding rate was calculated as the proportion of blood fed mosquitoes out of the total number of mosquitoes recaptured per hut each night. The data were log transformed for the blood-feeding rate.

#### Exit mortality

The proportion of dead mosquitoes at recapture was analysed with a linear mixed model that included insert treatment as independent variable. The night of capture and the hut were considered as random effects. The same analysis was conducted for the proportion of mosquitoes dead 24 h post recapture.

#### Deflection

To assess deflection the proportion of mosquitoes recaptured in the control hut was compared depending on the treatment in the adjacent hut (i.e., control, screening or treated eave tubes). Data were analysed with a linear mixed model that included the adjacent hut treatment as independent variable. The night of capture, the hut and the sleeper were considered as random effects.

An ANOVA was used to compare mortality at recapture and mortality 24 h post recapture between the different treatments inside the enclosure (control–control, control-screened, control-treated eave tubes).

#### Linear mixed models

For each experiment, the differences between treatments (whether the read outs were the mean number of mosquito entering a hut per night, or blood feeding, or dying) were analysed using analysis of variance incorporating random effects (these are designed to analyse the difference among group means in a sample). The analyses were done using the lme4 package, version 1.1.15, and the “lmer” function to obtain the linear mixed models in the software R version 3.5.0.

First the models were fitted and simplified for the random effects (like the night of capture or the hut). The likelihood ratio test (LRT) was used to compare models with or without the different random effects to see if these models are significantly different one from another. To do so, the “anova” function in the package lme4 was used, using the maximum likelihood method (ML) [17–20]. If a model with a given random effect was not significantly different from the same model without this random effect (p-value > 0.05) then the random effect was removed from the analysis.

The fixed effects (insert treatment or the type of opening in the hut) in the same linear mixed models were analysed using the restricted maximum likelihood (REML) approach. It was done using the package lme4, the package lmerTest, version 2.0-36, and the Kenward-Roger approximation [20–22]. The “anova” function of lmerTest package was used to perform the Kenward-Roger approximation. Fixed effects with p-values > 0.05 were considered not significant.

## Results

### Recruitment of mosquitoes to eave tubes

**Fig. 4:**
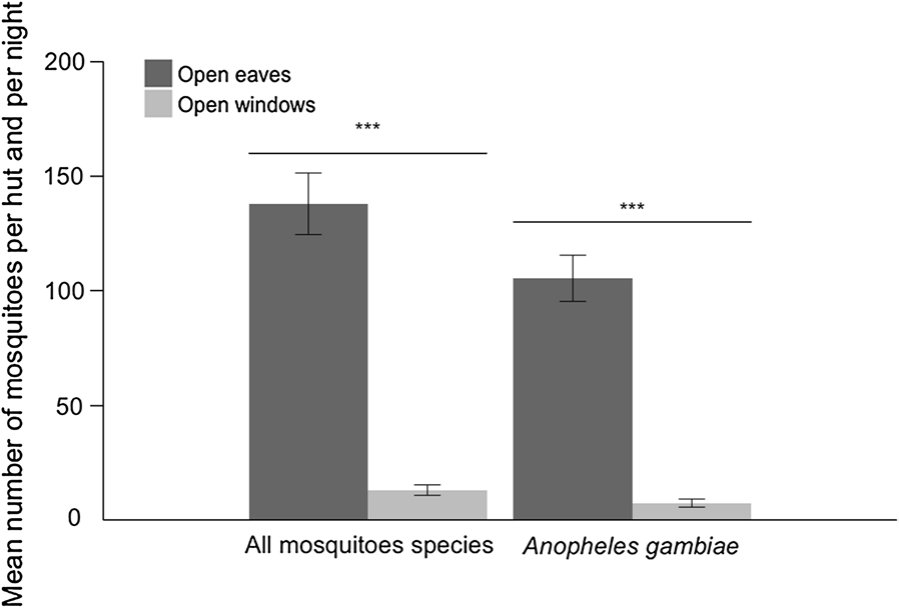
Mean (± SE) number of mosquitoes (all species) and of *Anopheles gambiae s.l.* captured per hut per night with open eaves tubes or open windows. The approach used 2 experimental huts that were assigned 1 of 2 treatments: (i) open eaves, in which eave tubes were open and windows closed; or (ii) open windows, in which eaves were closed and windows open. Means are based on a total of 20 nights of capture per treatment

About 93% more *An. gambiae* mosquitoes entered huts with open eaves (mean ± standard error (SE) = 105.4 ± 10.09) compared to hut with open windows (mean ± SE = 7.4 ± 1.77), (Fig. 4), (F_1,17_ = 133.46, p < 0.001). There was no effect of the hut or the night of capture (p > 0.05).

The preference for open eaves was about the same when all mosquito species were included, 90% more mosquitoes relative to open windows, F_1,17_ = 153.45 p < 0.001 (Fig. 4). There were mean ± SE = 138.0 ± 13.46 mosquitoes captured per hut and per night with open eaves and mean ± SE = 13.1 ± 2.29 with open windows.

Again, there was no effect of the hut or the night of capture (p > 0.05).

### Impact of eave tubes on mosquito entry

**Fig. 5:**
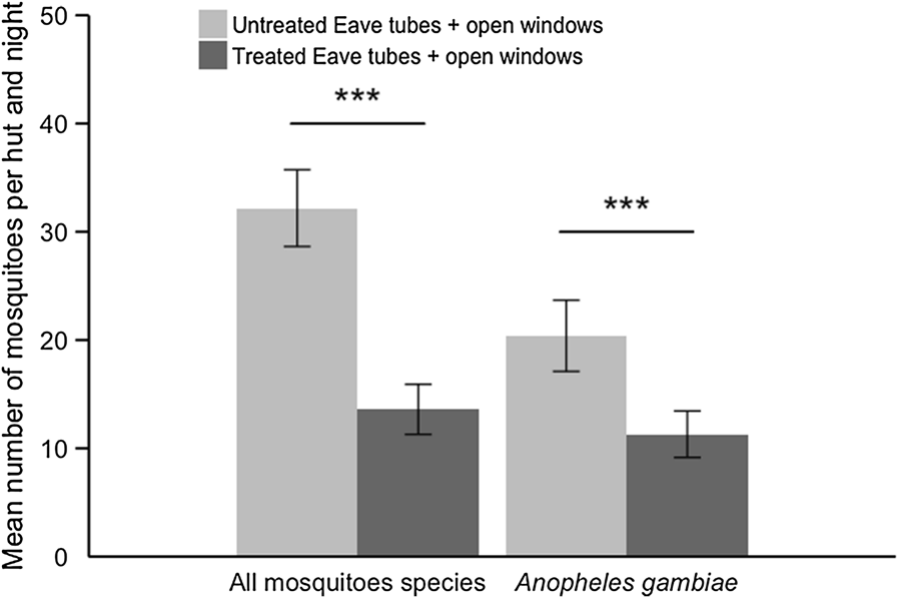
Mean (± SE) number of mosquitoes (all species) and of *Anopheles gambiae s.l.* captured per hut per night, comparing huts fitted with treated eave tubes or with untreated eave tubes. Both huts have open windows. Means are based on 24 nights of capture per treatment

Insecticide-treated eave tubes reduced entry of *An. gambiae* mosquitoes by 46% relative to control huts fitted with untreated eave tubes (F_1,23_ = 18.302, p < 0.001) (Fig. 5). There were mean ± SE = 11.0 ± 2.17 *An. gambiae* mosquitoes captured per hut and per night with insecticide-treated eave tubes and mean ± SE = 20.4 ± 3.29 *An. gambiae* mosquitoes captured with control huts fitted with untreated eave tubes. There was no effect of the hut nor the sleeper (both p > 0.05) but there was variation between the nights of capture (χ^2^ = 15.78, Chi.df = 1, p < 0.001).

The reduced entry rate was greater still when all mosquito species were included (60% reduction relative to controls, F_1,23_ = 47.53, p < 0.001) (Fig. 5). There were mean ± SE = 13.4 ± 2.33 mosquitoes captured per hut and per night with insecticide-treated eave tubes and mean ± SE = 32.2 ± 3.55 mosquitoes captured with control huts fitted with untreated eave tubes. Again, there were no significant effects of hut or sleeper (both p > 0.05), but some variation between nights (χ^2^ = 10.23, Chi.df = 1, p = 0.001).

**Fig. 6:**
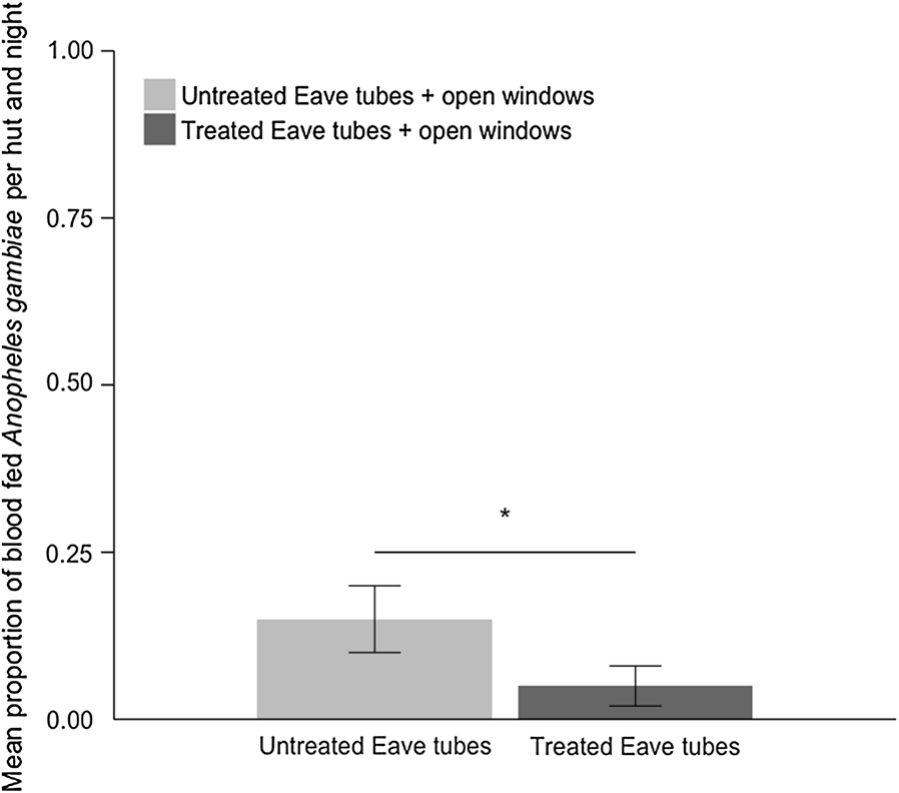
Mean (± SE) proportion of blood-fed *Anopheles gambiae s.l.* per hut and night comparing huts fitted with treated eave tubes or with untreated eave tubes. Both huts have open windows. Means are based on 24 nights of capture per treatment

In addition, treated eave tubes reduced blood feeding rate of mosquitoes that did manage to enter the huts by 64% (F_1,23_ = 4.49, p = 0.045) (Fig. 6). There were mean ± SE = 5.4 ± 2.66% of *An. gambiae* mosquitoes blood fed per hut and per night with insecticide-treated eave tubes and mean ± SE = 14.8 ± 4.59% of *An. gambiae* mosquitoes blood fed with control huts fitted with untreated eave tubes. There was no effect of the hut, the sleeper, or the night of capture (all p > 0.05).

Mosquitoes collected within the treated eave tube huts also showed higher mortality than those collected in the control huts at capture, mean ± SE = 23.2 ± 6.07% compared with mean ± SE = 12.6 ± 2.50% respectively, and 24 h post capture, mean ± SE = 31.4 ± 6.30% compared with mean ± SE = 19.1 ± 3.28% respectively. However, these mortality differences were not significant (F_1,22_ = 3.28, p = 0.083 for post-capture mortality and F_1,22_ = 3.64, p = 0.069 for 24 h post-capture mortality).

### Exit mortality

**Fig. 7:**
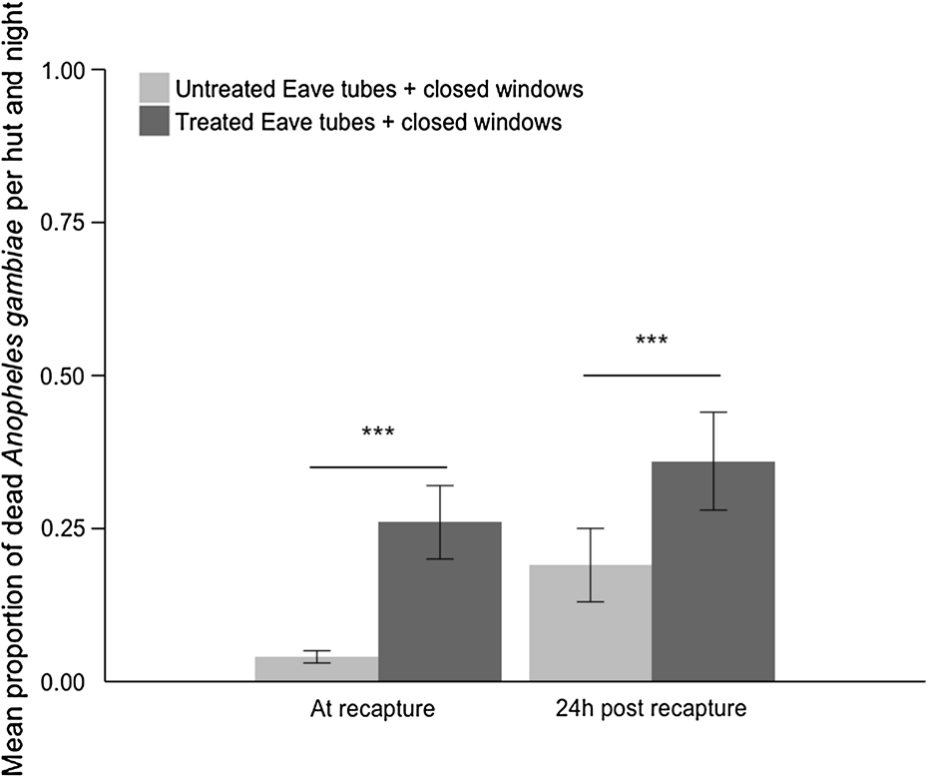
Effect of treated eave tubes on exit mortality. Adult *An. gambiae s.l*. were released into experimental huts with closed windows and door in the evening and recovered the following morning. The Figure shows the mean (± SE) proportion of dead mosquitoes at recapture or 24 h post recapture, comparing huts fitted with treated eave tubes, with huts fitted with untreated eave tubes. Treatments were replicated over 8 nights

Overnight mortality of mosquitoes released in a hut with treated eave tube inserts was significantly greater than mosquitoes released into a hut with untreated inserts (F_1,13_ = 14.16, p = 0.002), mean ± SE = 26.1 ± 6.08% and mean ± SE = 4.0 ± 0.60% respectively (Fig. 7). There was no effect of the hut or host on mortality (all p > 0.05).

Mortality of mosquitoes recovered from the huts and maintained for 24 h in the laboratory was also greater for the treated eave tube hut compared to the control hut (F_1,7_ = 34.79, p < 0.001), mean ± SE = 36.1 ± 7.60% and mean ± SE = 18.8 ± 5.80% respectively.

### Deflection between huts

On average, mean ± SE = 84.0 ± 1.80% of *An. gambiae* mosquitoes were recovered (alive or dead) following each release. The proportion of mosquitoes recruiting into huts within the enclosures was low. About 54% of mosquitoes were recaptured within the huts on nights when both huts were controls. This percentage reduced when one or other hut was screened or contained treated eave tubes.

**Fig. 8:**
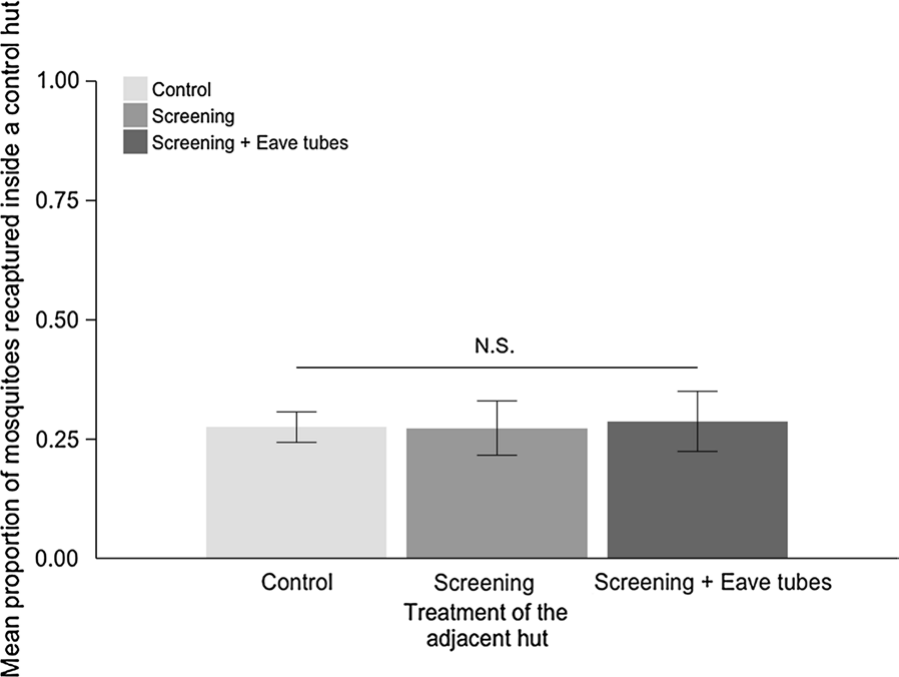
Mean (± SE) proportion of adult *Anopheles gambiae* captured inside a ‘control’ hut (i.e., a hut with open windows and open eaves) when paired with adjacent huts in a semi-field enclosure. Adult *An. gambiae s.l.* mosquitoes were released in the semi-field enclosure in the evening and recovered the following morning. Treatment pairings were control + control, control + screened hut (i.e., hut with untreated eave tube inserts and closed windows), and control + eave tubes (hut with treated eave tubes and closed windows). Means are based on 8 nights of release-recapture per treatment combination

The proportion of mosquitoes recaptured in the control huts was not influenced by the treatment of the adjacent hut (F_2,22_ = 0.13, P = 0.87); approximately 27% of mosquitoes released into the enclosure were recovered from inside an individual control hut regardless of which other hut it was paired with (i.e., another control, untreated eave tubes, or treated eave tubes) (Fig. 8).

The mean proportion of mosquitoes recaptured in the control hut when it was paired with another control hut was mean ± SE = 27.5 ± 3.26%. When the control hut was paired with a hut with screening and untreated eave tubes it was mean ± SE = 27.3 ± 5.70%. When the control hut was paired with a hut with screening and treated eave tubes it was mean ± SE = 28.8 ± 6.39%. There was a significant random effect of the night of release (χ^2^ = 6.07, Chi.df = 1, p = 0.013) but no effect of the hut or the sleeper (both p > 0.05).

**Fig. 9:**
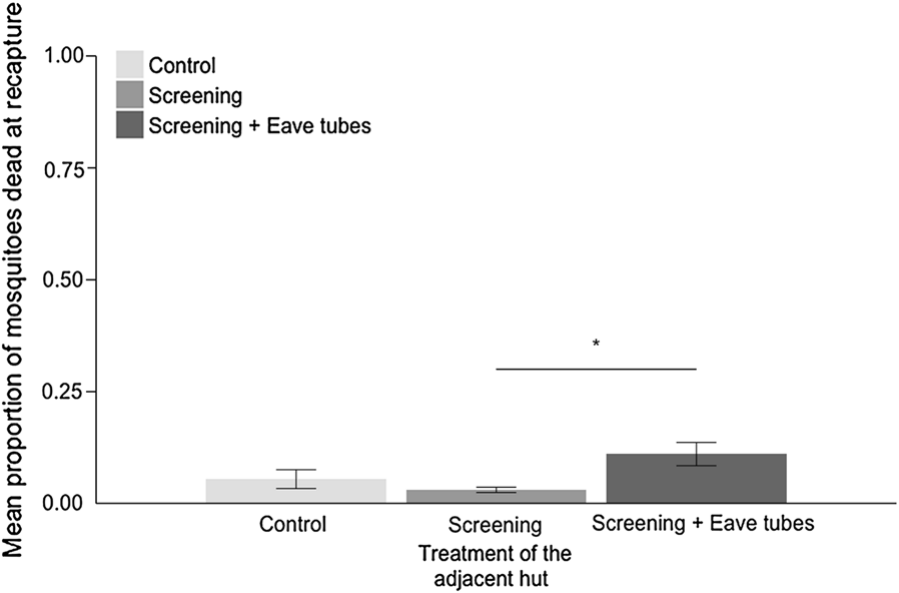
Mean (± SE) proportion of dead mosquitoes recovered from the semi-field enclosure for different treatment combinations. Adult *An. gambiae* (s.l.) mosquitoes were released in the semi-field enclosure in the evening and recovered the following morning. Here, ‘control’ indicates both huts had open eaves and open windows, ‘screening’ means 1 control hut and 1 with untreated eave tube inserts and closed windows, and ‘screening + eave tubes’ means 1 control hut and 1 with treated eave tubes and closed windows. Means are based on 8 nights of release-recapture per treatment combination

Overnight mortality was around mean ± SE = 3 ± 0.59% to mean ± SE = 5 ± 2.19% for combinations of control and screened huts (Fig. 9). There was a significant increase in mean mortality to mean ± SE = 11.0 ± 2.29% when treated eave tubes were added to one or other of the huts (F = 4.43, *df* = 2, p = 0.02). Given the expectation that around 27% of mosquitoes might have recruited to a hut with treated eave-tubes (this is the percentage that recruited to control huts, with no observed deflection), this mortality rate suggests that up to 40% of mosquitoes recruiting to an eave tube treated hut died in the enclosure overnight.

## Discussion

Previous studies suggest that eave tubes in combination with screening of doors and windows have the potential to reduce household entry of mosquitoes, increase mosquito mortality rate and reduce malaria transmission [4, 6, 7]. The results of the current experimental hut studies indicate that malaria vectors in Côte d’Ivoire actively recruit to eave tubes. The results also suggest that insecticide treated eave tubes alone can provide household level protection as they reduce mosquito entry even when no screening is present (i.e., window slits in the huts are open). The potential for eave tubes to reduce household entry of mosquitoes in the absence of screening is an important finding as it will likely be easier and cheaper to roll out eave tubes alone, compared with the combined package of eave tubes + screening. Those mosquitoes that did enter the huts exhibited reduced blood-feeding rate, suggesting an impact of sub-lethal contact with the insecticide-treated inserts as the mosquitoes initially sought to enter the hut via the eave tubes. Furthermore, it appears that mosquitoes can attempt to exit the huts through eave tubes providing an additional source of mortality (note however that the experimental huts were configured in such a way that mosquitoes were unable to exit the huts and so this might have increased encounter frequency with the eave tubes).

One of the potential concerns over eave tubes, and also household screening in general, is that mosquitoes that encounter a house that is difficult to enter might be deflected onto other houses that do not have any physical protection. This potential for deflection could undermine the utility of the intervention since it is extremely unlikely that coverage of houses will be 100% within a given location. Modelling studies exploring the effects of different levels of coverage of screening + eave tubes suggest that the impact of deflection is likely to be offset if there is increased mortality rate when mosquitoes encounter houses with eave tubes (i.e., a mass action effect should provide community wide protection reducing transmission risk even for those houses without the intervention) [7]. However, this prediction depends on the extent of deflection relative to mortality. The current study suggests that neither screening nor screening + eave tubes increases risk of deflection to non-treated huts. On the other hand, the addition of eave tubes to a hut more than doubles the overnight mortality rate of mosquitoes that attempt to enter that hut, reducing mosquito populations overall.

While the data are encouraging, it is important to acknowledge some limitations of the current study. First, the experiments were conducted using experimental huts, which are not the same as real houses. Whether the results hold up in real houses where open windows and doors potentially provide an easier route of entry and exit than the narrow window slits in the experimental huts is the subject of ongoing research.

Second, the deflection experiments were conducted in a large field cage and it is unclear whether this might have affected natural mosquito searching behaviour. The percentage of mosquitoes entering the huts was lower than expected (i.e., a maximum of 54% captured indoors when both huts were controls, meaning that about half the mosquitoes did not appear to recruit successfully). Nonetheless, an experimental hut study in The Gambia exploring deterrent effects of long-lasting insecticidal nets (LLINs) similarly found no evidence of deflection from houses with nets to adjacent houses without [23]. On the other hand, studies on topical repellents have suggested that under conditions of incomplete coverage, mosquitoes can be diverted from households that use repellent to those that do not [24].

Third, the mosquito release studies used young (4–5 days old), non blood-fed female mosquitoes reared from field-collected larvae. It is possible that wild mosquito populations of mixed condition, age, and infection status could exhibit different behaviour [25, 26], but there is no particular reason to think the current results are biased one way or the other.

Finally, experimental treatments using insecticides used freshly treated inserts with a maximum available dose of powdered insecticide (beta cyfluthrin). How patterns of mortality and effects of transient contact change over time as powder deposits decay in the field and/or inserts collect dust is currently being tested. Similarly, there are other possible active ingredients (including non-pyrethroids) and other potential delivery systems (for example, it might be possible to utilize LLIN coating technology or even a spot application with an insecticide spray to treat inserts inside the tubes) that could create opportunities for insecticide resistance management [27, 28], but these too require further testing.

## Conclusions

The data presented in the current study add weight to the potential for eave tubes to reduce malaria transmission. Important to note is that all semi-field experiments were conducted in the presence of LLINs and the malaria vectors at the study site are highly pyrethroid resistance [11–13]. The potential to bolster control above and beyond core control tools and deal with the challenge of insecticide resistance are important criteria for prospective vector control tools [2].

## Additional files

**Additional file 1.** Mosquitoes recruiting through eaves or windows. This data file gives the number of mosquitoes captured in each hut each morning depending on the hut treatment (open eaves or open windows) and the date. The sleeper is also noted.

**Additional file 2.** Impact of eave tubes on mosquito entry with open windows. This data file shows the number of mosquitoes captured in each hut each morning depending on the hut treatment (treated eave tubes or untreated eave tubes) and the date. The windows are open during the night. The hut location and the sleeper are also indicated.

**Additional file 3.** Exit mortality. Number of mosquitoes recaptured post release inside experimental huts. Mosquitoes alive, dead and/or blood fed at recapture and 24 h post recapture.

**Additional file 4.** Absence of deflection. Number of mosquitoes recaptured per control hut regarding the treatment of the adjacent hut.

**Additional file 5.** Mortality at recapture inside the enclosure. Number of dead mosquitoes at recapture in the enclosure depending on the treatments installed in the huts. The mortality 24 h post recapture is also available.

## Abbreviations

CRT: cluster randomized trial
IPR: Institut Pierre Richet
LLIN: long-lasting insecticide-treated net
LRT: likelihood ratio test
ML: maximum likelihood
PVC: polyvinyl chloride
REML: restricted maximum likelihood
WHO: World Health Organization
